# Estimating the Threshold Effects of Climate on Dengue: A Case Study of Taiwan

**DOI:** 10.3390/ijerph17041392

**Published:** 2020-02-21

**Authors:** Bao-Linh Tran, Wei-Chun Tseng, Chi-Chung Chen, Shu-Yi Liao

**Affiliations:** Department of Applied Economics, National Chung Hsing University, Taichung 402, Taiwan; linhtran.ymn@gmail.com (B.-L.T.); tweichun@nchu.edu.tw (W.-C.T.); mayjune@nchu.edu.tw (C.-C.C.)

**Keywords:** dengue, climate, threshold effect, vector index, negative binomial regression model

## Abstract

Climate change is regarded as one of the major factors enhancing the transmission intensity of dengue fever. In this study, we estimated the threshold effects of temperature on *Aedes* mosquito larval index as an early warning tool for dengue prevention. We also investigated the relationship between dengue vector index and dengue epidemics in Taiwan using weekly panel data for 17 counties from January 2012 to May 2019. To achieve our goals, we first applied the panel threshold regression technique to test for threshold effects and determine critical temperature values. Data were then further decomposed into different sets corresponding to different temperature regimes. Finally, negative binomial regression models were applied to assess the non-linear relationship between meteorological factors and Breteau index (BI). At the national level, we found that a 1°C temperature increase caused the expected value of BI to increase by 0.09 units when the temperature is less than 27.21 °C, and by 0.26 units when the temperature is greater than 27.21 °C. At the regional level, the dengue vector index was more sensitive to temperature changes because double threshold effects were found in the southern Taiwan model. For southern Taiwan, as the temperature increased by 1°C, the expected value of BI increased by 0.29, 0.63, and 1.49 units when the average temperature was less than 27.27 °C, between 27.27 and 30.17 °C, and higher than 30.17 °C, respectively. In addition, the effects of precipitation and relative humidity on BI became stronger when the average temperature exceeded the thresholds. Regarding the impacts of climate change on BI, our results showed that the potential effects on BI range from 3.5 to 54.42% under alternative temperature scenarios. By combining threshold regression techniques with count data regression models, this study provides evidence of threshold effects between climate factors and the dengue vector index. The proposed threshold of temperature could be incorporated into the implementation of public health measures and risk prediction to prevent and control dengue fever in the future.

## 1. Introduction

Dengue fever is a mosquito-borne communicable disease transmitted through the bite of female *Aedes* mosquito infected with dengue virus serotypes (DENVs 1–4) of the Flaviviridae family. The disease is currently considered the most widely distributed and rapidly spreading mosquito-borne viral disease in the world. Dengue fever has been now reported in over 124 countries and regions in the world, with an estimated 390 million DENV infections and 250,000 deaths occurring worldwide each year [1,2,3].

Many factors contribute to enhance of the transmission intensity of dengue. For example, increasing population, international tourism, global trading, and uncontrolled urbanization are considered important factors that could explain the rapid global spatial spread of dengue [4,5]. Additionally, regional expansion of the disease could be attributed to lack of public health infrastructure and inadequate knowledge about dengue fever [6,7]. In the context of these socioeconomic factors, the interactions between three spheres, namely human, mosquito, and virus factors in a given country, need to be associated with suitable weather and climate conditions before dengue fever can be established [8,9]. However, climate conditions do not directly influence the incidence of dengue. Instead, the larval development time, larval and adult survival, and duration of the gonotrophic cycle of the major dengue vector, *Ae. aegypti*, are directly affected by climactic factors such as ambient temperatures, rainfall, and relative humidity [10,11]. Many previous studies have indicated that meteorological parameters play an important role in increasing the risk of dengue transmission, depending on local ecology [12,13,14,15,16]. As there is no effective vaccine against dengue fever, dengue vector control remains the key measure for the prevention of *Ae.-aegypti*-transmitted diseases. Currently, the World Health Organization (WHO) recommends vector surveillance as a routine practice for predicting dengue outbreaks and evaluating disease control in dengue-endemic countries [17]. Using different statistical approaches such as regression coefficients, odd ratios, and rate ratios, many previous studies have provided evidence for positive correlations between vector indices (e.g., Breteau (BI), container (CI), and house indices (HI)) and human dengue cases [18,19,20]. However, some studies have concluded that there is no significant correlation between vector indices and dengue [21,22,23].

The relationship between dengue incidence and climate change has been extensively studied, but so far little attention has been paid to the threshold effects of climate on dengue vector indices. For policy purposes, it is important to construct a reliable alert system for checking and tracing whether a threshold effect of temperature on vector index exists in order to effectively reduce dengue risk. By monitoring the average temperature to see whether it has reached the threshold temperature or not, dengue fever prevention plans could be better prepared to avoid disease outbreaks. Therefore, in this study, we aimed to estimate the threshold effects of temperature on the Breteau index and to explore how this entomological index and the incidence of dengue fever are related in Taiwan. Our findings can provide valuable information for policy-makers in the implementation of public health measures to prevent and control dengue fever in the future. To fulfill our research goals, the specific objectives of the study were defined as (1) to examine the threshold effects of meteorological factors on a dengue vector index (BI); (2) to investigate the relationship between BI and the number of confirmed dengue cases; and (3) to explore the potential impacts of temperature on the BI dengue vector index under future climate change scenarios.

Evidence has shown that climate–dengue associations may vary within-country/-region or even within-province [9,13,24]. Therefore, our empirical models included a nationwide model and a southern Taiwan model, including Tainan, Kaohsiung, and Pingtung counties, for comparison purpose. By combining the panel threshold regression technique with count data models, we estimated the non-linear relationship between temperature and mosquito larval index (BI). To proceed, we first employed Hansen’s panel threshold model [25] to test for threshold effects and determine critical temperature values. Corresponding to different temperature regimes, data were then decomposed into different sets and negative binomial regression models were applied to assess the non-linear relationship between meteorological factors and BI. We also explored how the number of confirmed cases of dengue fever and vector surveillance data are related in Taiwan. Finally, climate change scenarios simulated by the Taiwan Climate Change Projection and Information Platform (TCCIP) were factored into the estimated coefficients to estimate the potential impacts of future climate change on the BI dengue vector index in Taiwan.

## 2. Background to Dengue Fever

### 2.1. Dengue Fever in Taiwan

Dengue fever is geographically distributed in tropical and subtropical regions. To date, most dengue outbreaks have been reported in countries of the Americas, South-East Asia, and Western Pacific regions [26]. The highest-risk zones are in Asia, representing about 70% of the total global burden of dengue disease, followed by Africa (16%) and the Americas (14%) [2]. In Taiwan, historical epidemics of dengue were documented in 1902, 1915, and 1922 in Penghu Islet; in 1924, 1927, and 1931 in southern regions; and in 1942–1943, spreading throughout the whole island of Taiwan [27]. As a result of worldwide programs for controlling malaria and dengue in simultaneously endemic areas after World War II (WWII), dengue fever was absent from Taiwan for some time after 1944 [28]. However, serious dengue outbreaks were recorded again in 1981 (13,000 reported cases) on the islet of Hsiao-Liu Chiu, Pingtung (Hsieh, 1982; Wu, 1986). After that, a DENV-1 epidemic occurred during the 1987–1988 period in southern Taiwan. Among 10,420 reported cases, the total number of indigenous cases was 5,336, with 241 cases of dengue hemorrhagic fever, including 19 deaths [27].

According to the computerized database of the surveillance system by Taiwan’s Center for Disease Control (Taiwan-CDC) [29], in the period of 2006 to 2012, there were 10,094 confirmed cases of dengue virus infections, or an average of about 1442 cases per year. However, from 2012 to 2018, the Taiwan-CDC recorded 63,471 confirmed cases of dengue, or an average of about 9067 cases per year. Thus, the annual average number of dengue cases has increased by 529% in the past seven years compared with the period from 2006 to 2012.

### 2.2. Dengue and Climate Change

Climate change is likely to expand the geographical distribution of several vector-borne human infectious diseases [30]. The risk of dengue transmission is increased by warming climates, as the growth and development of mosquitoes are significantly influenced by temperature and humidity [2]. Focks et al. [31] found that mosquito egg survival rate varied significantly when temperature is within the range of 22 to 34 °C. In another study conducted by Rowley and Graham [32], *Ae. aegypti* females were found to be sustainably able to fly between 15 and 32 °C. The optimal flight temperature, in terms of duration and distance flown, was found to be 21 °C, while the maximum flight speed (34.1 m/min) was recorded at 32 °C and 50% humidity. In addition to general activity and host-seeking behavior, favorable conditions for mosquitoes to initiate probing and blood-feeding have also been studied based on the difference between average environment temperature and host temperature (37 °C). Bishop and Gilchrist [33] reported a relatively high percentage of *Ae. aegypti* females imbibing blood at 42 °C, when the difference between average temperature of the environment and the blood meal was 14 °C (71%), than when the temperature of the blood and the environment were the same. The lower temperature limit at which *Ae. aegypti* has been found to cease biting is 15 °C and temperature at which they are the most active is 28 °C [34].

Ambient temperature and rainfall are also important factors that directly affect the development of dengue virus in major mosquito vectors *Ae. aegypti* and *Ae. albopictus*. Climate change will not only affect the rate of mosquito development, but also the virus incubation time [35]. In other words, climatic factors influence dengue ecology both directly and indirectly by affecting mosquito growth dynamics, virus replication, and mosquito–human interactions [9].

### 2.3. Quantitative Studies on Climate–Dengue Relationship

Many studies have found associations between climate conditions and the transmission of dengue. According to their findings, temperature, rainfall, and relative humidity have been identified as the most important climate variables related to the transmission of dengue. Additionally, mosquito larval indices have been widely used to predict the transmission of dengue. For example, Hwang [36] explored the relationship between *Aedes* mosquitoes and dengue fever epidemics in Taiwan in 1988–1990. The distribution and density of two different types of *Aedes* (*aegypti* and *albopictus*) were assessed in his study. The results indicated that the larval and adult density of *Aedes aegypti* and the larval density of *Ae. albopictus* are correlated with temperature, rainfall, and relative humidity. Increases in *Aedes* density were positively correlated to increases in temperature and rainfall. The peak of the *Aedes* density was also directly related to rainfall. Furthermore, the number of dengue fever cases was significantly correlated with seasonal population fluctuations and the regional density of *Ae. aegypti*.

It was found that the number confirmed cases during the 1987–1988 dengue outbreak in Taiwan was positively correlated with the Breteau index of *Ae. aegypti* [37]. Tseng et al. [38] studied how meteorological variables, Breteau index, and reported cases of dengue fever were related in Taiwan from January 2000 to February 2006. They tried to investigate how climate conditions influence mosquito density level at the 1st stage, and then estimated the relationship between dengue fever cases and density level in the 2nd stage. They found that climate conditions have significant impacts on dengue. For instance, when the temperature increased by 1%, the number of dengue patients in Kaohsiung and Pingtung increased by 5.75 and 11.83%, respectively.

Chen et al. [39] used a generalized additive model (GAM) which allowed Poisson regression to be fitted as a sum of a nonparametric smooth function of predictor variables to estimate the linear relationship between precipitation and dengue fever in Taiwan during the 1994–2008 period. They found that time-lagged effects following precipitation up to 350 mm were significantly correlated with increased risk of dengue.

Fan et al. [40] conducted a meta-analysis of 33 qualified articles to access the dengue risk associated with temperature change at a global level. Multiple linear regression and multivariate Poisson models were used in the study. Their results indicated that there is a positive correlation between the temperature and dengue. They concluded that the dengue incidence will increase by 35% per 1 °C increase while the average temperature is between 23.2 and 27.7 °C.

Xiang et al. [15] examined the temperature–dengue relationship in Guangzhou, China during the 2005–2014 period by using a piecewise linear spline function. The study found that the optimal temperature for transmission of dengue fever is 21.6–32.9 °C. Dengue cases started to increase when the temperature exceeded 21.6 °C, and then dropped dramatically when the temperature exceeded 32.9 °C. They also found that relative humidity was negatively correlated with dengue transmission when the daily relative humidity exceeded 79%.

Some recent studies have also included spatial component in modeling climate-related spread of dengue. For example, Yu et al. [41] proposed a spatiotemporal dengue fever prediction approach based on Bayesian maximum entropy analysis to estimate the climatic effects on dengue distribution in southern Taiwan. Applying a space–time Poisson process based on the surveillance data obtained for the 2002–2006 period, they found significant positive correlations between rainfall, minimum temperature, and dengue incidence. The predicted spatiotemporal dengue fever distribution was also very close to the actual distribution of dengue cases reported for the year 2007.

In addition to climate conditions, population density and urbanization are also considered important driving factors for dengue transmission. For example, Tseng et al. [38] indicated that population density has a positive and significant impact on the number of reported dengue cases for most counties in Taiwan. In addition, Gubler [5] claimed that rapid population growth in tropical urban areas often provides ideal ecological conditions for *Ae. aegypti* numbers to increase.

In general, different analytical approaches are applied depending on the distributional assumptions (e.g., Poisson, normal) and the spatial and/or temporal dynamics of the response. While many previous studies have assumed that the relationship between climate conditions and dengue transmission is linear in their models, a few studies, including Wu et al. [16], Descloux et al. [42], Chien and Yu [43], and Bultó et al. [44], concluded that the relationship between climate and dengue should be non-linear. Therefore, we also took non-linear relationships between climate and dengue into account when estimating the potential threshold effects of climate factors on dengue vector index. Our findings can provide important policy implications for the implementation of public health measures and risk prediction to prevent and control dengue fever in the future [15,16].

## 3. Estimating the Threshold Effects of Temperature on Dengue Vector Index

### 3.1. Dataset

The panel dataset covered the period from January 2012 to May 2019 for 17 counties and islets, including surveillance data on dengue case, entomological indices, and data on meteorological and population density. Below is a brief description of the dataset:

*Dengue case surveillance data:* In Taiwan, dengue fever is classified as a notifiable infectious disease and suspected cases must be reported to a clinic for diagnosis within 24 hours. Probable cases are defined as patients with body temperature above 38 °C and with least two of the following dengue-related clinical symptoms: rash, retro-orbital pain, leukopenia, myalgia, arthralgia, and hemorrhagic manifestations. In Taiwan, the dengue case surveillance system includes active surveillance (e.g., fever infrared thermal screening at airports) and passive surveillance (e.g., hospital-based reporting systems) for the comprehensive and effective surveillance of dengue infection. Epidemiological surveys of confirmed cases are conducted by local primary health centers. Suspected cases are confirmed by detecting dengue virus and differentiating virus serotypes using laboratory diagnosis. Nucleic acid identification of the dengue virus is identified by reverse-transcriptase (RT) polymerase chain reaction (PCM) (one-step real-time RT-PCR), serological testing on single or paired serum samples by dengue-specific envelope and membrane specific immunoglobulin M (IgM) and IgG antibody-capture enzyme-linked immunosorbent assay (with the exclusion of Japanese encephalitis virus infection), or virus isolation [45]. In this study, we used weekly confirmed dengue fever cases, which included indigenous cases and imported cases, obtained from the web-based National Infectious Disease Statistics System [29] under the Notifiable Disease Surveillance System (NDSS) of the Taiwan Center for Disease Control.

*Entomological surveillance data*: Since the *Stegomyia* indices are considered one of the most important measurements for the monitoring of dengue vector populations [17], we used the Breteau index (BI), which is the number of containers positive for *Ae. aegypti* larvae and pupae per 100 houses, as the dependent variable in our model. Surveillance of dengue-fever-carrying mosquito populations has been set up since the dengue outbreak in the southern counties of Taiwan in 1988. For all counties and cities, vector surveillance activities including mosquito species distinction, mosquito habitat recognition, and vector sampling are conducted by trained staff following the guidelines recommended by the World Health Organization [1]. Each community is considered a surveying unit (e.g., one unit in Kaohsiung County including 50–100 households was randomly selected for inspection [46]). Based on the household density and the average number of households positive for *Ae. aegypti* (from historical entomological data), the risk level of each surveying unit is determined. Furthermore, larval, pupae, and adult vector surveys for monitoring vector density, distribution, and breeding habitats are conducted for both indoor and outdoor areas. Depending on whether the surveying unit is classified as a high-, medium-, or low-risk area, the according inspection frequency will be weekly, monthly, or bi-monthly, respectively. The relevant surveillance data were retrieved from Taiwan National Infectious Disease Statistics System, Centers for Disease Control [47]. As the inspection date and time varied across cities and townships in Taiwan, the data extracted included a huge combination of daily average BI values. We reorganized the county-level BI dataset and converted daily data into weekly data to ensure the consistency of start-day and end-day of each week in the dengue surveillance data.

*Meteorological data*: The meteorological variables included daily mean temperature, daily mean accumulative rainfall, and daily mean relative humidity. The data were systematically retrieved from the Central Weather Bureau (CWB), Taiwan [48]. We calculated the average values of available weather data from different stations in each county. Daily weather data were then aggregated to weekly data.

*Others*: Other explanatory variables included population density data, regional total population, and total area data, which were retrieved from the Department of House Registration, Ministry of Interior (MOI), Taiwan.

Descriptive statistics of these variables are shown in Table 1.

### 3.2. Estimating Temperature Thresholds

Temperature is widely considered the most important climatic factor for dengue incidence prediction, as it plays a more critical role in dengue transmission than other meteorological factors [13,39,49]. Therefore, we focused on examining the non-linear relationship between dengue vector index and temperature in this study. To search for two or more regimes endogenously, Hansen’s [25] threshold model was employed to test whether or not there exist threshold effects between BI and temperature.

Following Hansen (1999), the structure of the single-panel threshold model used was as follows:

(1) $$y_{it}=u_{i}+\beta_{1}^{\prime }x_{it}I\left(q_{it}\leq \gamma \right)+\beta_{2}^{\prime }x_{it}I\left(q_{it}>\gamma \right)+e_{it}$$

where the data are from a balanced panel; *i* and *t* denote indices of the individual (1≤i≤N) and time (1≤t≤T), respectively; *y_it_* and the threshold variable *q_it_* are scalars; *x_it_* is a *k* vector of explanatory variables; I(·) is an indicator function; $u_{i}$ is the fixed effect (or heterogeneity of individuals); and the error term $e_{it}$ is assumed to be independent and identically distributed, $e_{it}\sim iid\left(0,\;\sigma^{2}\right)$. Equation (1) can be rewritten as follows.

(2) $$y_{it}=u_{i}+\beta^{\prime }x_{it}\left(\gamma \right)+e_{it}$$

where $\beta^{\prime }x_{it}\left(\gamma \right)=\{\begin{array}{c} \beta_{1}^{\prime }x_{it}I\left(q_{it}\leq r\right)\\ \beta_{2}^{\prime }x_{it}I(q_{it}>r) \end{array}$.

The data were separated into two regimes, whereby the threshold variable $q_{it}$ was less than or greater than the threshold value γ. The two regimes had different regression slopes $\beta_{1}^{\prime }$ and $\beta_{2}^{\prime }$, respectively.

Averaging Equation (2) over time led to

(3) $${\bar{y}}_{it}=u_{i}+\beta^{\prime }{\bar{x}}_{i}\left(\gamma \right)+{\bar{e}}_{i}$$

where ${\bar{y}}_{i}=1/T\sum_{t=1}^{T}y_{it},\;{\bar{x}}_{i}=1/T\sum_{t=1}^{T}x_{it},\;\mathrm{and}\;{\bar{e}}_{i}=1/T\sum_{t=1}^{T}e_{it}$.

Subtracting Equation (3) from (2) led to

(4) $$y_{it}^{*}=\beta^{\prime }x_{it}^{*}\left(\gamma \right)+e_{it}^{*}$$

or, in vector form

$$y_{i}^{*}=\left[\begin{array}{c} y_{i2}^{*}\\ \vdots \\ y_{iT}^{*} \end{array}\right],\;\;x_{i}^{*}\left(\gamma \right)=\left[\begin{array}{c} x_{i2}^{*}\left(\gamma \right)\\ \vdots \\ x_{iT}^{*}\left(\gamma \right) \end{array}\right],\;and\;e_{i}^{*}=\left[\begin{array}{c} e_{i2}^{*}\\ \vdots \\ e_{iT}^{*} \end{array}\right]$$

We stacked the data over individuals into Y^*^, X^*^, and e^*^, and then derived Equation (5) to estimate threshold effects.

(5) $$Y^{*}=\left[\begin{array}{c} y_{1}^{*}\\ \vdots \\ y_{N}^{*} \end{array}\right],\;\;X^{*}\left(\gamma \right)=\left[\begin{array}{c} x_{1}^{*}\left(\gamma \right)\\ \vdots \\ x_{N}^{*}\left(\gamma \right) \end{array}\right],\;and\;e^{*}=\left[\begin{array}{c} e_{1}^{*}\\ \vdots \\ e_{N}^{*} \end{array}\right]$$

$$Y^{*}=\beta^{\prime }X^{*}\left(\gamma \right)+e^{*}.$$

Ordinary least-squares (OLS) method was used to estimate β for a given γ.

(6) $$\hat{\beta }\left(\gamma \right)={\left(X^{*}\left(\gamma \right)\prime X^{*}\left(\gamma \right)\right)}^{-1}X^{*}\left(\gamma \right)\prime Y^{*}.$$

The vector of regression residuals was

(7) $$\hat{e}\left(\gamma \right)=Y^{*}-X^{*}\left(\gamma \right)\hat{\beta }\left(\gamma \right),$$

which was minimized for SSE to estimate γ:

(8) $$SSE_{1}\left(\gamma \right)={\hat{e}}^{*}\left(\gamma \right)\prime {\hat{e}}^{*}\left(\gamma \right)$$

where

(9) $$\hat{\gamma }=arg\;min\;SSE_{1}\left(\gamma \right)$$

The estimated slope coefficient was $\hat{\beta }=\hat{\beta }\left(\hat{\gamma }\right)$, the vector of residuals was ${\hat{e}}^{*}={\hat{e}}^{*}\left(\hat{\gamma }\right)$, and the estimated variance of the residuals was

(10) $${\hat{\sigma }}^{2}=\frac{1}{N\left(T-1\right)}{\hat{e}}^{{*}^{\prime }}{\left(\hat{\gamma }\right)}^{\prime }{\hat{e}}^{*}\left(\hat{\gamma }\right)=\frac{1}{N\left(T-1\right)}SSE_{1}\left(\hat{\gamma }\right).$$

Supposing a single threshold effect was found between temperature and entomological surveillance index BI, the empirical panel threshold model was as follows.

(11) $$BI_{it}=\mu_{i}+\alpha_{1}Temp_{it-\tau }I\left(Temp_{it-\tau }\leq \gamma \right)+\alpha_{2}Temp_{it-\tau }I\left(Temp_{it-\tau }>\gamma \right)+\;\alpha_{3}Precp_{it-\tau }+\alpha_{4}Humidity_{it-\tau }+\varepsilon_{it}$$

For a balanced panel, i and t denote province and time (week), $BI_{it}$ is entomological surveillance (Breteau) index, $Temp_{it}$ is average temperature (℃), $Precp_{it}$ is average precipitation (mm), *Humid_it_* is relative humidity (%), τ represents the time lag, and $\varepsilon_{it}$ is the error term. Since there is about 2 weeks from laying to hatching of eggs, and eggs will hatch into larvae within 24 to 48 hours, a 2 week lag (τ = 2) was chosen to estimate the effects of meteorological factors on mosquito larval index BI.

Before estimating Equation (11), we applied panel unit root tests to examine whether the variables were stationary or not, and the results indicated they were stationary (please refer to Appendix A). Equation (11) was estimated to see whether there were one, two, or three thresholds. Table 2 displays the results of the threshold effect tests, including the test statistics F_1_, F_2_, F_3_ and their corresponding bootstrap *p*-values.

According to the results of the nationwide model, a single threshold effect F_1_ was statistically significant at about the 5% level (*p*-value = 0.06), while the tests for a double F_2_ and a triple threshold F_3_ were not significant. This indicated that a single temperature threshold exists at the nationwide level. Based on the results of the Southern model, a single threshold F_1_ was statistically significant at the 1% level and a double threshold F_2_ was also statistically significant at the 1% level, while the test result of triple thresholds F_3_ was not significant. We concluded that there are two temperature thresholds in the southern region of Taiwan. The estimates of the temperature thresholds are reported in Table 3.

**Figure 1:**
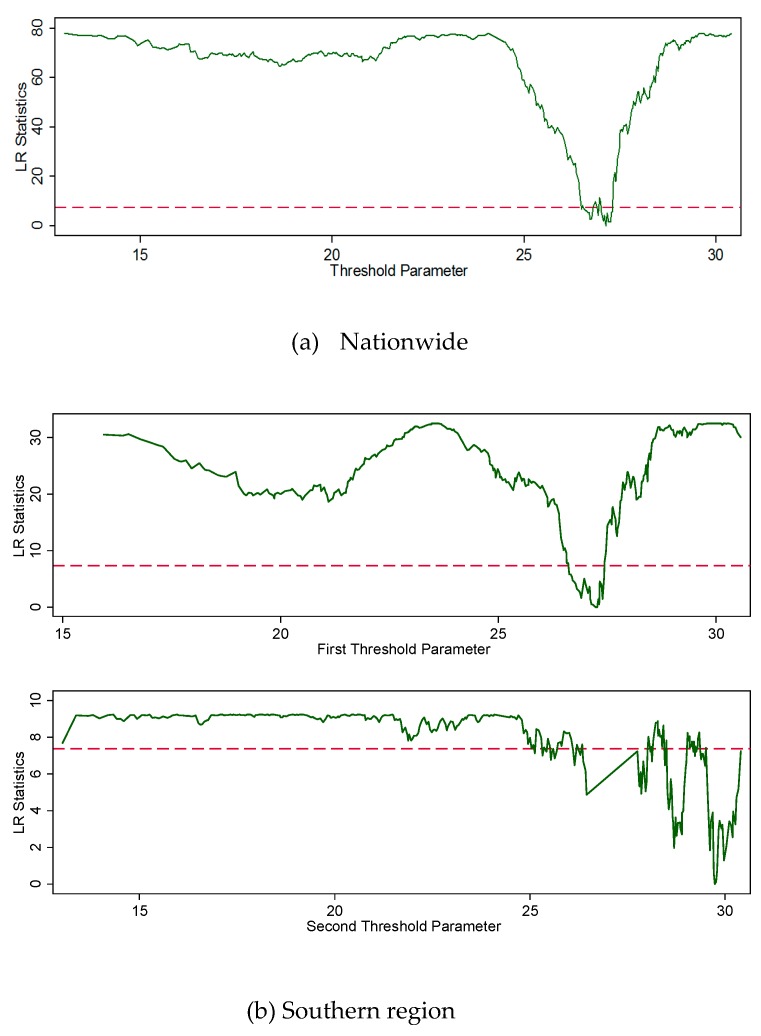
Confidence interval construction for (a) Nationwide (single) and (b) Southern region (double) thresholds. Note: The dash lines denote the critical value (7.35) at the 95% confidence level.

Table 3 shows that the estimated single threshold was 27.21 °C at national level. For the double threshold effects of temperature in the Southern region, the estimated values were 27.27 and 30.17 °C. The likelihood ratio (LR) statistics are plotted in Figure 1 to display the confidence interval construction for the nationwide model (a) and the southern region model (b).

### 3.3. Estimating Threshold Effects of Meteorological Factors on Breteau Index

After estimation of the temperature thresholds of the BI, this study went one step further to examine the threshold effects of weather factors on the dengue vector index. In this stage, count data regression models were employed because the dependent variable (*BI_it_)* was a non-negative integer random variable. Most previous studies have applied Poisson regression models to estimate the relationship between ecological factors and dengue. However, the Poisson model has a strong restriction in that the variance and mean are equal, an assumption which is often violated in real datasets. When the conditional variance exceeds the conditional mean, the count data are over-dispersed. As a consequence, hypotheses on the Poisson regression parameters may be rejected more often than they should be [50,51], since the estimation also includes underestimated standard errors of parameter estimates. To resolve this issue, we first described dengue cases by the corresponding BI, so the means of dengue cases within each level of BI were lower than the variances within each level. In other words, the conditional means were lower than the conditional variances. We then fit two regression models specifically developed for count outcomes, Poisson and negative binomial (NB), and then compared these two models using the likelihood ratio (LR) test.

The LR test performs a test of the null hypothesis that the parameter vector of a statistical model satisfies some smooth constraints. To conduct the test, both the unrestricted and the restricted models must be fit using the maximum likelihood. Let L_0_ and L_1_ be the log-likelihood values associated with the full (NB) and constrained (Poisson) models, respectively. The test statistic of the likelihood ratio test is LR = −2(L_1_ -L_0_). If the constrained model is true, LR is approximately χ^2^ distributed with *d_0_ – d_1_* degrees of freedom, where *d_0_* and *d_1_* are the degrees of freedom associated with the full and constrained models, respectively [52]. The LR test statistic is approximately distributed as chi-squared, and was separately established for the nationwide model and the southern region model.

As can be seen in Table 4, the results of the test statistic allowed us to reject the constrained model hypothesis for both the nationwide model and the southern region model. Table 4 also includes the model selection indices including Akaike’s information criterion (AIC) and the Bayesian information criterion (BIC). The results indicated that the NB regression model was favored over the Poisson for estimation in both the nationwide and southern region models.

To estimate the threshold effects of weather factors on BI dengue vector index using panel NB regression models, we decomposed data into separate sets according to the single temperature threshold in the nationwide model and double temperature thresholds in the southern region model. The effects of meteorological factors on BI were estimated as follows.

For the nationwide model:

(12) $$\begin{array}{ll} E\left(BI_{it}|X_{it},\;\epsilon_{it}\right)= & \exp\;(\alpha_{10}+\alpha_{11}Temp_{it-\tau }+\alpha_{12}Precp_{it-\tau }+\alpha_{13}Humid_{it-\tau }+\epsilon_{it})I(Temp_{it-\tau }\\ & \leq 27.21\;℃) \end{array}$$

(13) $$\begin{array}{ll} E\left(BI_{it}|X_{it},\;\epsilon_{it}\right) & =\exp(\alpha_{20}+\alpha_{21}Temp_{it-\tau }+\alpha_{22}Precp_{it-\tau }+\alpha_{3}Humid_{it-\tau }+\epsilon_{it})I(Temp_{it-\tau }\\ & >27.21\;℃) \end{array}$$

For the southern region model:

(14) $$\begin{array}{ll} E\left(BI_{it}|X_{it},\;\epsilon_{it}\right) & =\exp(\beta_{10}+\beta_{11}Temp_{it-\tau }+\beta_{12}Precp_{it-\tau }+\beta_{13}Humid_{it-\tau }+\varepsilon_{it})I(Temp_{it-\tau }\\ & \leq 27.27\;℃) \end{array}$$

(15) $$\begin{array}{ll} E(BI_{it}|X_{it},\;\epsilon_{it}) & =\exp(\beta_{20}+\beta_{21}Temp_{it-\tau }+\beta_{22}Precp_{it-\tau }+\beta_{23}Humid_{it-\tau }\\ & +\varepsilon_{it})I\left(27.27\;℃<Temp_{it-\tau }\leq 30.17\;℃\right) \end{array}$$

(16) $$\begin{array}{ll} E\left(BI_{it}|X_{it},\;\epsilon_{it}\right) & =\exp(\beta_{30}+\beta_{31}Temp_{it-\tau }+\beta_{32}Precp_{it-\tau }+\beta_{33}Humid_{it-\tau }+\varepsilon_{it})I(Temp_{it-\tau }\\ & >30.17\;℃) \end{array}$$

The NB parameter was assumed to follow a Gamma distribution. The NB model was considered a generalization of Poisson model, since it had the same mean structure as Poisson regression and it had an extra parameter to model the over-dispersion (i.e., error term $\varepsilon_{it}$ allowed the conditional variance of y to exceed the conditional mean). We also computed marginal effects by multiplying the estimated coefficients with the exponential of expected value of the dependent variable BI. The estimation results are displayed in Table 5.

At the national level, when the weekly average temperature was less than 27.21 °C, a 1 °C increase in average temperature caused the expected value of BI to increase by 0.09 unit. When the weekly average temperature was higher than 27.21°C, a 1 °C increase in temperature led to a 0.26 unit increase in the expected value of BI. For the southern regions of Taiwan, the impacts of all weather factors on dengue vector index were stronger than those at the national level. Specifically, when the weekly average temperature was less than 27.27 °C, a 1 °C increase in temperature led to a 0.29 unit increase in the expected value of BI. When the weekly average temperature was between 27.27 and 30.17 °C, the expected value of BI increased by 0.63 units for every additional degree Celsius increase. When the weekly average temperature was above 30.17 °C, a 1 °C increase in temperature caused the expected value of BI to increase by 1.49 units.

The empirical results also indicated that the impacts of precipitation and relative humidity on dengue vector index vary under different regimes of weekly average temperature. At the national level, relative humidity had a negative effect on BI when weekly average temperature was less than 27.21 °C. However, this effect became positive when weekly average temperature exceeded 27.21 °C. For the southern region of Taiwan, the effects of relative humidity on the expected value of BI became stronger when temperature increased. There were significant positive effects of precipitation on the expected value of BI when weekly average temperature was below 30.2 °C. However, the effects of precipitation on the expected value of BI became insignificant when the weekly average temperature rose above 30.2 °C.

## 4. Estimating the Relationship between Entomological Index and Dengue Cases

Because the number of confirmed cases of dengue fever was considered count data, a similar process of estimating panel count data models was applied as in the previous section. We used LR tests to examine which regression model was more appropriate for our data set and found that the negative binomial model was preferred. The results of the LR tests are shown in Table 6.

The empirical NB regression model can be writtern as follows:

(17) $$E\left(DF_{it}|X_{it},\;\epsilon_{it}\right)=\exp(\beta_{0}+\beta_{1}BI_{it-\tau }+\beta_{2}Pop\_den_{it}+\epsilon_{it})$$

For a balance panel, *i* and *t* denote county and time (week), $DF_{it}$ is the number of confirmed cases of dengue fever, $BI_{it}$ is the Breteau index, $Pop\_den_{it}$ is the population density, τ represents the time lag, and $\varepsilon_{it}$ is the error term. In this study, the lagged effect of BI on dengue cases was also considered. We employed a 4 week lag to take into account the mosquito life cycle from larvae to adult flying mosquitoes (2 weeks), and the incubation periods of dengue viruses after mosquito feeding on an DENV-infected person (2 weeks) [53,54]. The estimated results are displayed in Table 7.

The estimation results showed that a positive relationship existed between the incidence of dengue and the 4 week lagged BI. We also found a positive relationship between the number of dengue cases and the population density. The estimated incidence rate ratios (IRR) indicated that both BI and population density had higher effects on the incidence of dengue in the southern region of Taiwan than nationwide.

## 5. Projecting the Effects of Temperature on Entomological Index under Climate Change Scenarios

To investigate the potential effects of future climate change on the dengue vector index, temperature projections were retrieved from the Taiwan Climate Change Projection Information and Adaptation Knowledge Platform (TCCIP). This future climate change projection is the simulation of future possible climate scenarios in Taiwan at a 5 × 5km resolution for the 2021–2100 period with a baseline period of 1886–2005. A a set of four representative concentration pathways (RCPs) was employed in this study: RCP2.6, RCP4.5, RCP6.0, and RCP8.5, corresponding to radiative forcing levels of 2.6, 4.5, 6.0, and 8.5 watts per square meter, respectively.

The projections for the temperature change associated with the four RCP scenarios are shown in the first row of Table 8. Projected temperatures are equal to these projected temperature changes plus the weekly average temperature in 2012–2019. Depending on whether the projected average temperature exceeded the threshold values (27.21 °C for the nationwide and 27.27 °C and 30.17 °C for the southern region) or not, we calculated the potential effects of the changes in temperature on the expected value of BI by multiplying the projected changes in temperature with the corresponding estimated marginal effects of temperature on BI. The percentage changes of BI were also computed by multiplying the change in expected value of BI by 100 and then dividing by the average BI.

At the nationwide level, the increase in BI peaked at 5.30% in the 2041-2060 period in the RCP 2.6 scenario. For the RCP 4.5 scenario, the increase in BI peaked at 8.87% in the 2081–2100 period. The BI reached a 11.18% increase in the 2081–2100 period in the RCP 6.0 scenario. For the RCP 8.5 scenario, the BI increase reached 17.75% in the 2081-2100 period. Under future climate change scenarios, average temperatures in the southern region exceeded the threshold level of 27.27 °C in the RCP 6.0 scenario during the 2081–2100 period (27.35 °C). For the RCP 8.5 scenario, average temperatures exceeded the threshold level of 27.27 °C during the periods of 2061–2080 (27.71 °C) and 2081–2100 (28.47 °C). Under the RCP 8.5 scenario, values of BI were predicted to increase more dramatically in the southern region than at the nationwide level, peaking at 40.77% and 54.54% during the periods of 2061–2081 and 2081–2100, respectively.

## 6. Discussion

It is widely recognized that there exists a positive correlation between the increase of vector indices and the increasing occurrence of dengue epidemics [18,19,20]. In this study, we not only obtained consistent findings in quantifying the relationship between entomological index (BI) and dengue incidence, but also estimated the non-linear effects of climate factors on an entomological index (BI) under different threshold regimes of temperature.

In this study, we found one temperature threshold (27.21 °C) at the nationwide level and two temperature thresholds (27.27 °C and 30.17 °C) in the southern region of Taiwan. Our estimation result is consistent with previous findings regarding the ideal climate conditions for mosquito growth dynamics. Specifically, female mosquitoes will survive better than the male mosquitoes when the temperature rises to 25 °C [14] and the maximum female mosquito survival rate is about 88–93% between 20 and 30 °C [55]. Moreover, the estimated temperature threshold of about 27 °C was close to Connor’s finding [34] that *Ae. aegypti* was most active around 28 °C. A recent study by Wu et al. [16] also examined the non-linear effect of temperature on dengue incidence and identified a single temperature at 28 °C. All the temperature thresholds found in this study also fell within the range of optimal temperature for the mosquito egg to survive (22–34°C) [31] and the range of temperature at which mosquitoes can best fly (15–32°C) [32].

In addition to estimating the threshold effect of temperature on a dengue vector index (BI), we also investigated the relationship between BI and dengue incidence. For every additional BI increase with 4 weeks lagged time, the number of confirmed dengue cases was found to increase correspondingly, with a roughly equivalent degree of the effects at the national level and the southern regional level. The coefficient estimates of BI were positive and significant at both national and southern regional levels. Therefore, our findings were consistent with previous studies and can provide evidence to support using this vector index to predict the risk of dengue in practice [13,20,46].

## 7. Conclusions

### 7.1. Contributions of the Study

Although the interactions between climate factors, dengue vector indices, and dengue cases have been extensively studied, the potential threshold effects of climate factors on dengue vector indices had not previously been estimated in Taiwan. Our empirical results indicated that the effects of temperature on dengue vector index (BI) will differ depending on the temperature levels. At the national level, when the weekly average temperature was less than 27.21 °C, an increase of 1 °C in temperature caused a 0.09 unit increase in the expected value of BI. The effect became stronger when the temperature exceeded 27.21 °C, with the expected value of BI increasing by 0.26 units when the temperature increased by 1 °C. Additionally, the effects of temperature on dengue vector index in the southern region of Taiwan were found to be stronger than those at the national level. As the temperature increased by 1 °C, the expected value of BI increased by 0.29, 0.63, and 1.49 units when the average temperature was less than 27.27 °C, between 27.27 and 30.17 °C, and higher than 30.17 °C, respectively.

According to the estimation results, we concluded that dengue fever infection rates in regions with warmer temperatures will be much more sensitive to climate change. Compared with the entire nation, such a difference in regional climate sensitivity was reflected in the double thresholds of temperature found in the southern region. The effects of other climatic factors, including precipitation and relative humidity, on BI also became stronger when the temperature exceeded these thresholds. This further emphasizes the importance of finding potential temperature thresholds in the implementation of public health measures and risk prediction to prevent and control dengue fever.

On the assumption that other factors affecting dengue cases remain the same, we also found that increases of Breteau index resulted in substantial increases in the risk of dengue fever. Investigating the potential effects of future climate change on the dengue vector index, we found that increases in projected temperature caused the value of BI to increase, ranging from a low value of 3.51% to a high value of 17.75% at the national level. For the southern region of Taiwan, the impacts were much stronger, with increases in the value between 5.13 and 54.42%. This implies that southern regions will be more vulnerable to climate-change-induced dengue fever and hence more effects should be devoted to reducing the risk in this region.

### 7.2. Implications of the Study

Extreme changes in weather and climate events containing extreme temperature events are highly likely to increase in both frequency and strength in the future. It is important to identify potential temperature thresholds for dengue vector indices in order to quantify the effects of temperature on climate-related spread of dengue and better support early warning systems for dengue epidemics. Therefore, our research findings can provide valuable information for future epidemiological studies investigating the relationship between climatic factors and dengue fever incidence.

In Taiwan, the major prevention strategies for the control of dengue fever are devised to eliminate vector (mosquito) breeding sources and lower vector density. The identification of specific temperature thresholds could be utilized to effectively support these dengue prevention measures. In particular, identification of temperature thresholds is important to construct a reliable alert system not only for disease prevention personnel but also for every resident. When average temperature is close to the threshold value, household and surrounding environment sanitation should be strengthened to eliminate all potential vector breeding sources. When temperature continues to increase and exceeds the threshold value, integrated strategies and practices for dengue prevention and control (e.g., environmental approaches to eliminate container habitats; chemical, biological, and genetic approaches; and personal actions regarding dengue prevention interventions) are urgently needed, especially in high-risk communities/regions like the southern region of Taiwan.

### 7.3. Limitations of the Study and Eecommendations for Further Research

Although we identified potential threshold effects of climate factors on dengue, our analysis had several limitations. First, due to the lack of long-term data on meteorological and other socio-ecological changes, the empirical estimates of climate effects on dengue may change when more data become available in the future. Second, dengue cases may be underestimated due to asymptomatic infection cases, and the quality of vector surveillance data may also vary county by county due to differences in the procedure and effort investing in the surveillance. As a result, empirical estimates at the national level may be less accurate than those at regional levels. Finally, our empirical analysis was based on weekly county-level panel data, which did not effectively take the geographical dimension into account due to the lack of detailed dengue surveillance data. However, research on estimating the effects of climate factors on dengue should not be limited to the analysis of panel data. Spatiotemporal approaches with more detailed dengue surveillance data could better describe the spatial spreading and transmission patterns of dengue epidemic.

Future studies are recommended to develop models at regional scale with more detailed community-level data and spatial information. In addition, the methodology proposed in this study could also be utilized for other regions/countries to estimate the relationship between climatic factors, vector indices, and dengue incidence. This would be very useful to support an early warning system for dengue epidemics worldwide, because it has been found that the habitat range of dengue vectors has expanded from areas of low latitude to mid- or high-latitude regions as the temperature has increased. Therefore, future studies are encouraged to focus on the climate-related habitat expansion and spread of dengue vectors.

## Figures and Tables

**Table 1:** Descriptive statistics of variables used in analyses

Region		Dengue Fever (Cases)	BI	Temperature (℃)	Humidity (%)	Precipitation (mm)	Population Density (per km^2^)
Nationwide	Mean	9.65	1.51	23.457	77.06	5.59	1225.36
	St. Dev	118.81	1.93	4.77	6.78	10.47	2233.55
	Max	3416	30.44	32.56	98.86	123.14	9956.10
	Min	0	0	0	46.86	0	61.79
Southern	Mean	52.55	3.48	25.44	74.25	5.56	719.15
	St. Dev	278.95	3.16	3.77	5.73	12.99	299.13
	Max	3416	30.44	31.33	94.43	105.86	995.42
	Min	0	0	13.21	55.86	0	296.20

Note: St. Dev denotes standard deviation, Max denotes maximum value, Min denotes minimum value.

**Table 2:** Tests for threshold effects.

	Nationwide	Southern
Test for single threshold		
$F_{1}$	108.48	28.56
*p*-value	0.060	0.000
Critical values (10%, 5%, 1%)	92.34, 120.63, 153.91	15.83, 15.93, 17.37
		
Test for double threshold		
$F_{2}$	17.58	9.31
*p*-value	0.52	0.000
Critical values (10%, 5%, 1%)	34.83, 41.12, 54.98	7.40, 7.74, 8.04
		
Test for triple threshold		
$F_{3}$	18.76	7.21
*p*-value	0.45	0.92
Critical values (10%, 5%, 1%)	35.65, 43.37, 64.02	12.62, 12.74, 14.03

**Table 3:** Temperature threshold estimates.

Region	Threshold Effect	Estimates	95% Confidence Intervals
Nationwide	Single threshold	$\hat{\gamma }$ = 27.21	[27.09, 27.24]
Southern	Double threshold	${\hat{\gamma }}_{1}^{r}$ = 27.27	[26.92, 27.29]
		${\hat{\gamma }}_{2}^{r}$ = 30.17	[29.74, 30.19]

**Table 4:** Likelihood ratio test results.

Assumption: Poisson Nested in NB						
**Model**	**Test Statistic**	**Observation**	**LL (model)**	**df**	**AIC**	**BIC**
**Nationwide**						
Panel Poisson	LR-chi2(1) = 37.22 Prob > chi2 = 0.000	6545	−8680.7	5	17,352.55	17,389.49
Panel NB		6545	−8652.2	6	17,316.33	17,357.05
**Southern**						
Panel Poisson	LR-chi2(1) = 116.88 Prob > chi2 = 0.000	1155	−2489.1	5	4986.18	5006.39
Panel NB		1155	−2357.9	6	4727.49	4758.29

**Table 5:** Estimation results of the effects of weather factors on Breteau index.

Region	Temperature Range	Variable	Coefficient	Marginal Effect	Std. Err	95% CI
Nationwide	Temp ≤ 27.21	Temp.	0.069***	0.087	0.004	[0.060, 0.075]
		Precip.	0.006***	0.007	0.001	[0.003, 0.008]
		Humid.	−0.009***	−0.011	0.004	[−0.014, 0.005]
		Constant	−0.642***		0.202	[−1.041, −0.243]
	Temp > 27.21	Temp.	0.087***	0.261	0.017	[0.073, 0.143]
		Precip.	0.008***	0.023	0.001	[0.006, 0.010]
		Humid.	0.042***	0.122	0.003	[0.035, 0.047]
		Constant	−4.762***		1.023	[−6.783, −2.751]
Southern	Temp ≤ 27.27	Temp.	0.098***	0.288	0.011	[0.078, 0.119]
		Precip.	0.011***	0.032	0.002	[0.005, 0.016]
		Humid.	−0.005	−0.147	0.005	[−0.016, 0.006]
		Constant	−1.191***		0.455	[−2.085, 0.296]
	27.27 < Temp ≤ 30.17	Temp.	0.112***	0.625	0.006	[0.096, 0.122]
		Precip	0.006***	0.035	0.001	[0.002, 0.008]
		Humid.	0.024***	0.134	0.004	[0.014, 0.032]
		Constant	−3.431***		0.326	[−4.073, −2.789]
	**Temperature Range**	**Variable**	**Coefficient**	**Marginal Effect**	**Std. Err**	**95% CI**
	Temp > 30.17	Temp.	0.453**	1.487	0.208	[0.005, 0.801]
		Precip.	−0.004	−0.013	0.014	[−0.032, 0.024]
		Humid.	0.068***	0.225	0.019	[0.030, 0.106]
		Constant	−17.565**		7.506	[−32.297, −2.874]

Note: Std. Err. denotes standard errors and *, **, and *** denote significance at the 10%, 5%, and 1% levels, respectively.

**Table 6:** Likelihood ratio test results.

Assumption: Poisson Nested in NB						
Model	Test Statistic	Observation	LL (model)	df	AIC	BIC
**Nationwide**						
Panel Poisson	LR-chi2(1) = 306,944 Prob > chi2 = 0.000	6511	−160,726.3	4	321,460.7	321,487.8
Panel NB		6511	−7253.8	5	14,517.7	14,551.6
**Southern**						
Panel Poisson	LR-chi2(1) = 308,356	1149	−160,726.3	4	132,471.2	132,723.4
Panel NB	Prob > chi2 = 0.000	1149	−2986.2	5	5982.3	6007.5

**Table 7:** Estimation results for the effects of Breteau index and population density on dengue cases.

Variable	Coefficient	Std. Err.	Marginal Effect	IRR	95% CI
Nationwide					
BI	0.028***	0.008	0.013	1.028***	[1.008, 1.047]
Pop._Den.	0.001**	0.000	0.0005	1.000***	[0.999, 1.001]
Constant	−2.015***	0.049			
Southern					
BI	0.075***	0.009	0.016	1.077***	[1.056, 1.097]
Pop._Den.	0.001	0.000	0.0002	1.001***	[0.998, 1.002]
Constant	−2.515***	0.174			

Note: Std. Err. denotes standard errors and *, **, and *** denote significance at the 10%, 5%, and 1% levels, respectively.

**Table 8:** Percentage change in Breteau index under climate change projections.

Scenarios	Year	Nationwide				Southern			
		RCP 2.6	RCP 4.5	RCP 6.0	RCP 8.5	RCP 2.6	RCP 4.5	RCP 6.0	RCP 8.5
Temperature Change Projection	2021–2040	0.63 (2.69%)	0.67 (2.86%)	0.61 (2.60%)	0.77 (3.28%)	0.62 (2.44%)	0.66 (2.59%)	0.66 (2.59%)	0.76 (2.99%)
	2041–2060	0.92 (3.92%)	1.14 (4.86%)	0.93 (3.96%)	1.48 (6.31%)	0.90 (3.54%)	1.13 (4.44%)	0.92 (3.62%)	1.46 (5.74%)
	2061–2080	0.87 (3.71%)	1.43 (6.10%)	1.42 (6.05%)	2.30 (9.81%)	0.86 (3.38%)	1.41 (5.54%)	1.40 (5.50%)	2.27 (8.92%)
	2081–2100	0.77 (3.28%)	1.54 (6.57%)	1.94 (8.27%)	3.08 (13.1%)	0.76 (2.99%)	1.52 (5.98%)	1.91 (7.51%)	3.03 (11.9%)
Change in Expected Value of BI	2021–2040	0.05	0.06	0.05	0.07	0.18	0.19	0.18	0.22
	2041–2060	0.08	0.10	0.08	0.13	0.26	0.33	0.26	0.42
	2061–2080	0.08	0.12	0.12	0.20	0.25	0.41	0.40	1.42
	2081–2100	0.07	0.13	0.17	0.27	0.22	0.44	1.19	1.89
Percentage Change in BI	2021–2040	3.63	3.86	3.51	4.44	5.13	5.46	5.13	6.29
	2041–2060	5.30	6.57	5.36	8.53	7.45	9.35	7.61	12.08
	2061–2080	5.01	8.24	8.18	13.25	7.12	11.67	11.59	40.77
	2081–2100	4.44	8.87	11.18	17.75	6.29	12.58	34.30	54.42

## Appendix A

**Table A1:** Panel unit root test results, using Levin–Lin–Chu test, Im–Pesaran–Shin test and Fisher-PP test at I(0).

Variables	LLC Test	IPS Test	PP-Fisher Chi-Sq.
Breteau Index (BI)	−47.58***	−54.06***	131.73***
Dengue Cases (DF)	−28.81***	−48.65***	118.41***
Average Temperature (Temp)	−2.49**	−13.27***	27.93***
Precipitation (Precp)	−72.06***	−66.60***	144.49***
Relative Humidity (Humid)	−48.37***	−47.13***	140.77***

Note: table displays the results of panel unit root tests where ** and ***, respectively, denote significance at the 5% and 1% levels.
